# The Impact of Fine Particulate Matter on Embryonic Development

**DOI:** 10.3390/ijms25126399

**Published:** 2024-06-10

**Authors:** Chia-Ta Wu, Ting-Shuan Wu, Min-Sho Ku

**Affiliations:** 1 School of Medicine, Chung Shan Medical University, No. 110, Sec. 1, Chien-Kuo N. Road, Taichung 402, Taiwan; allenpig1102@gmail.com; 2 Department of Emergency Medicine, Changhua Christian Hospital, Changhua 500, Taiwan; 3 Department of Biomedical Sciences, Chung Shan Medical University, Taichung 402, Taiwan; 4 Division of Allergy, Asthma and Rheumatology, Department of Pediatrics, Institute of Allergy, Immunology, and Rheumatology, Chung Shan Medical University Hospital, No. 110, Sec. 1, Chien-Kuo N. Road, Taichung 402, Taiwan

**Keywords:** air pollution, fine particulate matter, inflammatory responses, pathway

## Abstract

Airborne fine particulate matter (PM2.5) in air pollution has become a significant global public health concern related to allergic diseases. Previous research indicates that PM2.5 not only affects the respiratory system but may also induce systemic inflammation in various tissues. Moreover, its impact may vary among different populations, with potential consequences during pregnancy and in newborns. However, the precise mechanisms through which PM2.5 induces inflammatory reactions remain unclear. This study aims to explore potential pathways of inflammatory responses induced by PM2.5 through animal models and zebrafish embryo experiments. In this study, zebrafish embryo experiments were conducted to analyze the effects of PM2.5 on embryo development and survival, and mouse experimental models were employed to assess the impact of PM2.5 stimulation on various aspects of mice. Wild-type zebrafish embryos were exposed to a PM2.5 environment of 25–400 μg/mL starting at 6 h after fertilization (6 hpf). At 6 days post-fertilization, the survival rates of the 25, 50, 100, and 200 µg/mL groups were 100%, 80, 40%, and 40%, respectively. Zebrafish embryos stimulated with 25 μg/mL of PM2.5 still exhibited successful development and hatching. Additionally, zebrafish subjected to doses of 25–200 μg/mL displayed abnormalities such as spinal curvature and internal swelling after hatching, indicating a significant impact of PM2.5 stimulation on embryo development. In the mouse model, mice exposed to PM2.5 exhibited apparent respiratory overreaction, infiltration of inflammatory cells into the lungs, elevated levels of inflammatory response-related cytokines, and inflammation in various organs, including the liver, lungs, and uterus. Blood tests on experimental mice revealed increased expression of inflammatory and chemotactic cytokines, and GSEA indicated the induction of various inflammatory responses and an upregulation of the TNF-α/NFκB pathway by PM2.5. Our results provide insights into the harmful effects of PM2.5 on embryos and organs. The induced inflammatory responses by PM2.5 may be mediated through the TNF-α/NFκB pathway, leading to systemic organ inflammation. However, whether PM2.5-induced inflammatory responses in various organs and abnormal embryo development are generated through different pathways requires further study to comprehensively clarify and identify potential treatment and prevention methods.

## 1. Introduction

Air pollutants refer to one or more substances present in the atmosphere, including particulate matter, gases, and chemicals. These pollutants originate from various human activities and natural phenomena. The concentration, quantity, and duration of exposure to air pollutants may pose health hazards to humans [1]. The primary route of contact and exposure to air pollutants is through inhalation into the respiratory system. These pollutants may lead to cell inflammation, oxidative stress, immune responses, gene mutations, and the onset of diseases [1,2].

The World Health Organization (WHO) has identified air pollution as a significant factor severely affecting human health. Not only is it classified as a carcinogen, but it also poses a high risk and impact on individuals with allergies [1]. According to a 2013 study by the International Agency for Research on Cancer (IARC), particulate matter is listed as one of the primary environmental factors causing cancer-related deaths [3]. A more recent WHO report indicates that approximately 7 million people die each year due to indoor and outdoor air pollution, with a strong correlation between air pollutants and cardiovascular diseases, acute and chronic respiratory diseases, and cancer [1].

Our previous studies [4,5] and others [6,7,8,9] suggest a close correlation between air pollutants, especially particulate matter (such as PM2.5 and PM10), gases (such as nitrogen dioxide and ozone), and the generation and exacerbation of various diseases. Diseases induced by exposure to air pollutants include respiratory system disorders such as chronic obstructive pulmonary disease (COPD), bronchitis, and asthma [1]; cardiovascular diseases such as heart disease, stroke, and heart failure [10]; and neurological disorders such as brain damage, stroke, Alzheimer’s, and Parkinson’s disease [11], as well as potential impacts on child development and fetal health [12,13].

In our daily lives, we unavoidably inhale or come into contact with particulate matter through both the nasal cavity and skin. While most particulate matter can be cleared by mechanisms such as nasal mucosal cilia and the skin barrier, PM2.5, with a diameter smaller than 2.5 μm, can linger within lung tissues upon inhalation or contact. It can breach the respiratory barrier, gaining access to the circulatory system and causing systemic diffusion. Consequently, a growing body of research suggests that PM2.5 not only influences respiratory organs but also potentially affects the health of diverse tissues [14].

Previous studies [10,14,15] have pointed to several potential health effects that PM2.5 may have, such as increasing the incidence of diabetes and cardiovascular diseases [10,14]. Concerning the adverse effects of PM2.5 exposure on pregnancy, it has been proposed that exposure during gestation could disrupt the endocrine balance in pregnant women [15]. Concerning the adverse effects of PM2.5 exposure on pregnancy, it has been proposed that exposure during gestation could disrupt the endocrine balance in pregnant women. This disruption may affect the delivery of oxygen and nutrients to the fetus through the placenta, potentially resulting in preterm birth and low birth weight [15]. Additionally, prenatal exposure to PM2.5 may lead to impaired neurodevelopment in newborns [16].

Other studies [17,18] have demonstrated that maternal exposure to PM2.5 during pregnancy could impact placental growth factor binding, consequently affecting placental function. Moreover, prenatal exposure to PM2.5 has been linked to an elevated risk of childhood asthma and a potential association with neonatal jaundice induction, as indicated by our prior investigation [19].

Even with low PM2.5 exposure levels, potential health risks persist. A prior study [20] suggested that PM2.5 can induce inflammation and elevate oxidative stress levels in the body, resulting in damage to proteins, lipids, and even deoxyribonucleic acid (DNA). However, the exact mechanisms underlying the effects of PM2.5, oxidative stress, and respiratory inflammatory diseases still require further elucidation.

An experimental study [21] observed that exposure to PM2.5 triggers inflammatory responses, alters gene expression, and enhances protein secretion in both human and animal cells in a dose-dependent manner. This results in elevated levels of proinflammatory cytokines such as tumor necrosis factor-alpha (TNF-α), interleukin-1 beta (IL-1β), intereukin-6 (IL-6), interleukin-8 (IL-8), and monocyte chemoattractant protein-1 (MCP-1) [22,23]. Additionally, PM2.5 exposure has been associated with a significant increase in eosinophils and elevated levels of albumin, urea, and α1-antitrypsin in asthmatic children [24].

Exposure to PM2.5 has also demonstrated an increase in macrophage infiltration and the upregulation of pro-inflammatory genes such as TNF-α, MCP1, and leptin in mice [25]. Although one study [26] suggests that PM2.5 exposure may reduce the expression of toll-like receptor 4 (TLR4) or toll-like receptor 2 (TLR2), type 2 T helper (Th2)-related cytokines like interleukin-4 (IL-4), interleukin-5 (IL-5), interleukin-10 (IL-10), and interleukin-13 (IL-13) were found to remain significantly elevated. However, type 1 T helper (Th1) cytokines such as interferon gamma (IFN-γ), which balance Th2 responses, showed a decrease [27]. Therefore, exposure to PM2.5 is likely to cause changes in the immune system.

Moreover, exposure to PM2.5 may activate the nuclear factor kappa-light-chain-enhancer of activated B cells (NF-κB) pathway by phosphorylating nuclear p65 and cytoplasmic inhibitory-κB kinase-α (IKK-α). This can lead to the dose-dependent binding of nuclear p65/p50 DNA complexes in human lung epithelial cells, triggering inflammatory responses and potentially causing abnormalities in embryonic development [28,29]. One study also indicates that exposure to PM2.5 in children may lead to elevated levels of various inflammatory biomarkers in the bloodstream [30]. Furthermore, the NF-κB activation induced by PM2.5 and its associated inflammatory responses could potentially be prevented proactively.

We have encountered additional studies on PM2.5 [31,32,33,34,35,36,37], which highlight its undeniable impact on the respiratory system and lungs [35]. Concerningly, its effects on childrens’ health might outweigh those observed in adults [33,36]. Moreover, there is evidence suggesting that PM2.5 exposure can induce chronic inflammation in women, potentially disrupting ovarian function [37]. This poses risks not only to fertility but also to the health of newborns born to affected women [33]. Regarding cardiovascular diseases, exposure to PM2.5 may worsen existing conditions or precipitate their onset [34]. Additionally, some research underscores the adverse effects of PM2.5, whether it infiltrates through food contamination or indoor work environments, on human health [31,32].

Viewing this comprehensively, exposure to PM2.5 extends its influence beyond specific organs, potentially triggering systemic inflammation. The impact of PM2.5 on diseases is broad and varied, with potentially more severe effects on specific demographics, including the elderly, pregnant women, children, individuals with respiratory allergies, and asthma patients [38,39].

Nonetheless, further investigation is imperative to elucidate the precise exposure levels or PM2.5 components responsible for severe inflammation.

To improve the effectiveness of preventive and interventional measures in both medical and preventive healthcare, it is crucial to conduct comprehensive research and analysis into the mechanisms by which air pollutants induce different diseases. This study aims to utilize embryo experiments and animal models to further explore the pathway analysis of PM2.5-induced systemic inflammation. The goal is to provide more accurate insights and guidelines for preventing and managing PM2.5-induced diseases in the future.

## 2. Results

### 2.1. Survival Analysis of Zebrafish Embryos Exposed to PM2.5

**Figure 1:**
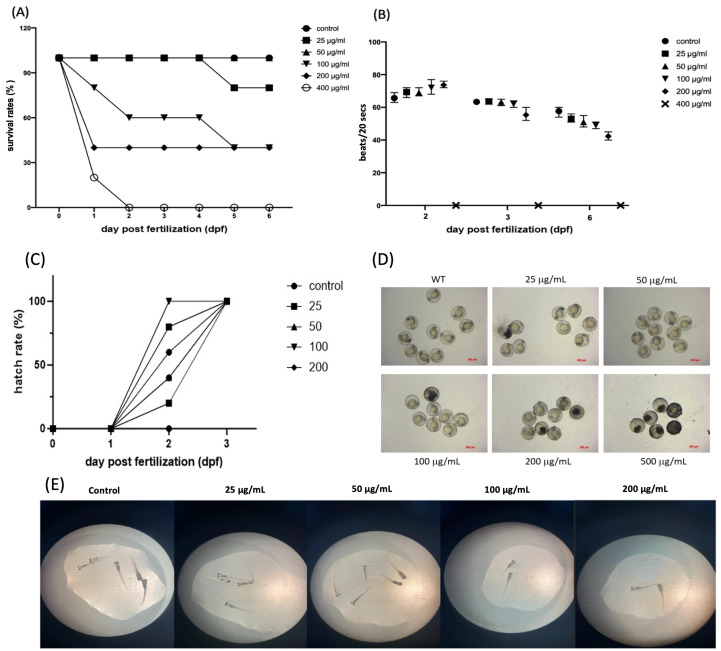
Illustration of the results of observing the survival rate of zebrafish embryos after exposure to PM2.5. (**A**) illustrates the embryo survival rate six days post-exposure, (**B**) shows the heartbeat count during embryo development, (**C**) presents the embryo hatch rate, (**D**) presents the appearance of zebrafish embryos under different dosage treatments, and (**E**) depicts the morphology of zebrafish after successful hatching.

Figure 1 illustrates the condition of zebrafish embryos under PM2.5 stimulation, including survival status, heart development, hatch rate, and overall morphology. Zebrafish embryos at 6 hpf were exposed to various PM2.5 concentrations to determine the optimal concentration and exposure time for subsequent experiments. Wild-type zebrafish embryos were exposed to PM2.5 concentrations ranging from 25 to 400 µg/mL. The survival rate was observed until 6 days post-fertilization (dpf). As shown in Figure 1A, embryos exposed to 400 µg/mL all died by 2 dpf, while at 6 dpf, the survival rates for the 25, 50, 100, and 200 µg/mL groups were 100%, 80%, 40%, and 40%, respectively. Embryos exposed to 25 µg/mL did not show any mortality during the experiment. However, embryos treated with concentrations greater than 25 µg/mL demonstrated lower heartbeats, spinal curvature, or death at the stage of 6 dpf (Figure 1B,D). The hatch rate in each group did not show a significant decrease (Figure 1C). Despite successful hatching, zebrafish exposed to PM2.5 stimulation exhibited some morphological abnormalities, such as spinal curvature (Figure 1E).

### 2.2. Effect of PM2.5 in Mouse Model

**Figure 2:**
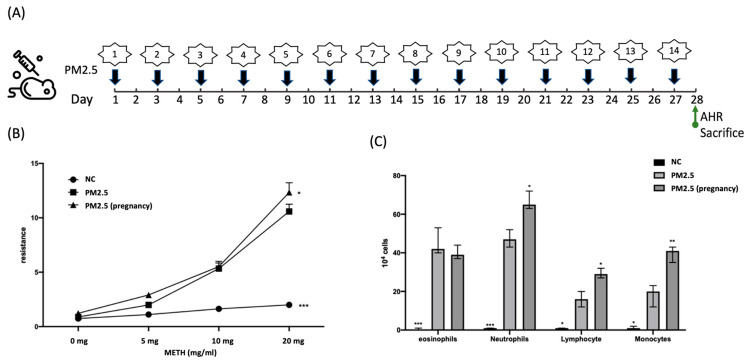
Illustration of the results of PM2.5 stimulation in wild-type BALB/c mice and pregnant mice. (**A**) outlines the experimental procedure for PM2.5 stimulation in mice. (**B**) displays respiratory resistance measurements, and (**C**) presents various inflammatory cell counts in the bronchoalveolar lavage fluid (BALF) of experimental mice. Statistical analysis compared all data with the group exposed only to PM2.5. Asterisks denote significance levels as follows: * indicates a *p*-value less than 0.05, ** indicates a *p*-value less than 0.01, and *** indicates a *p*-value less than 0.001.

Based on these results, exposure to PM2.5 indeed induces respiratory symptoms in experimental mice (Figure 2). From the airway resistance measurements in Figure 2B, it can be observed that exposure to PM2.5 leads to a significant increase in airway resistance, with a more pronounced effect observed in the pregnant group. The cell count results from the bronchoalveolar lavage fluid (BALF) in Figure 2C reveal a significant increase in inflammatory cells in the airways. It is worth noting that the increase in neutrophil count appears to be higher than that of eosinophils, suggesting that the inflammatory response triggered by airway pollutants may differ from that caused by typical allergens.

Airway hyperresponsiveness testing is based on clinical testing methods, including measuring respiratory values after inhaling different concentrations of methacholine.

### 2.3. Effect of PM2.5 on Gene Expression of Th1/Th2/Th17/Treg in Mouse Model

**Figure 3:**
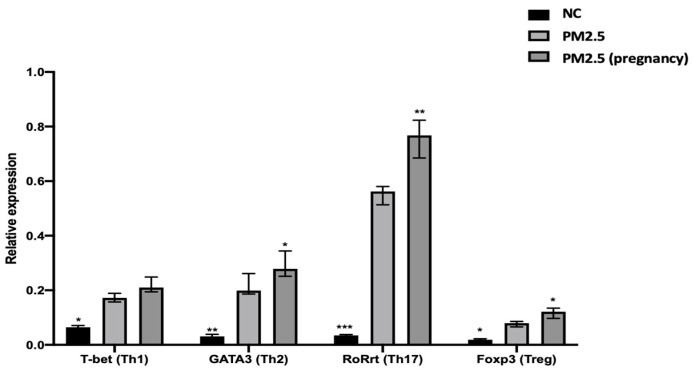
Depiction of the PCR identification analysis of Th1/Th2 and Th17/Treg in the lungs of wild-type BALB/c mice and pregnant mice after exposure to PM2.5. Statistical analyses were conducted, comparing all data with the PM2.5-exposed group. Asterisks denote significance levels as follows: * indicates a *p*-value less than 0.05, ** indicates a *p*-value less than 0.01, and *** indicates a *p*-value less than 0.001.

Figure 3 presents the polymerase chain reaction (PCR) analysis performed on the fresh lung tissues collected from experimental mice. PCR analysis revealed significantly upregulated expression of both GATA binding protein 3 (GATA3) and RAR-related orphan receptor-γ (RoRγt) in mice exposed to PM2.5 stimulation, regardless of pregnancy status. Pregnant mice in the experimental group notably demonstrated markedly higher expression of the RoRγt gene associated with T helper 17 cells (Th17) compared to other groups. In the pregnant mouse group stimulated by PM2.5, regulatory T (Treg) cells also exhibited higher expression levels. These results suggest that different populations undergo distinct inflammatory responses following exposure to PM2.5 stimuli.

### 2.4. Effect of PM2.5 on Inflammation in Various Tissues

**Figure 4:**
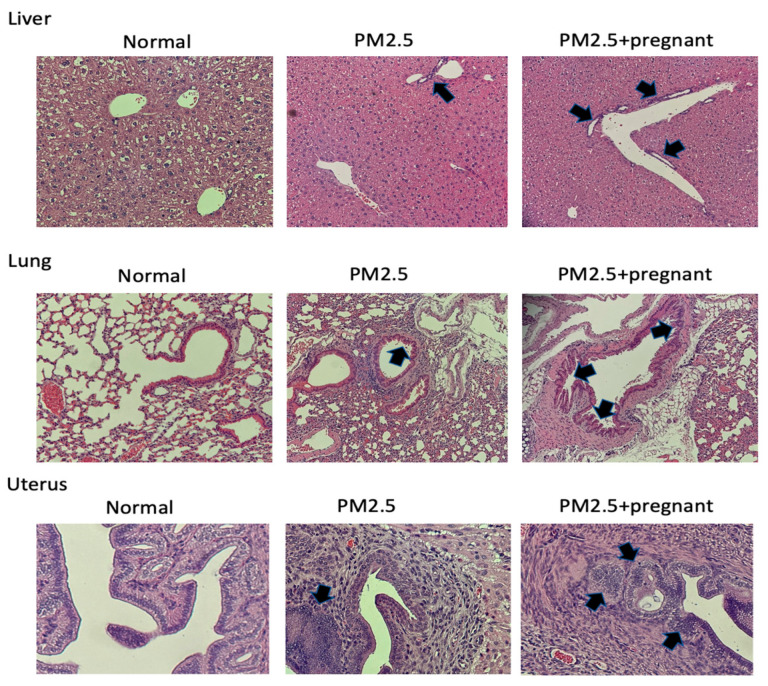
Display of the histological sections stained with hematoxylin and eosin (H&E) of the lungs, liver, and uterus of experimental mice. According to these research findings, it is evident that PM2.5 significantly induces inflammation in various tissues of the experimental mice.

Figure 4 shows the liver, lung, and uterus tissues collected and processed into pathological sections. These sections were stained with H&E to assess tissue inflammation. In the liver, compared to the normal control (NC) mice and non-pregnant mice stimulated with PM2.5, pregnant mice showed a more pronounced inflammatory condition, as indicated by black arrows. In the lung, airway inflammation was evident with H&E staining. Pregnant mice exposed to PM2.5 exhibited significantly more severe goblet cell hyperplasia and airway inflammation compared to non-pregnant mice exposed to PM2.5. In the uterus, H&E staining revealed signs of inflammation under PM2.5 stimulation. Additionally, sections of the uterus from pregnant mice displayed multiple areas of inflammatory conditions.

### 2.5. GSEA of PM2.5 in Animal Model

Gene set enrichment analysis (GSEA) was used to analyze the impact of PM2.5 on various cytokine and chemokine indicators in mice. Pathways identified include INFLAMMATORY_RESPONSE and TNFA_SIGNALING_VIA_NFKB. The summarized potential participating biological markers for each pathway are detailed in Table 1. In this part, a cytokine array assay was employed to assess the peripheral blood from mice by testing approximately 111 cytokines and chemokines. Subsequently, GSEA was conducted to deduce factors influencing the observed effects.

## 3. Discussion

Based on our established air pollution animal experimental model, we observed various inflammatory conditions, confirming the success of this model. The research results indicate that the inflammatory response induced by ambient PM2.5 may differ among different populations.

Furthermore, in the experimental model involving pregnant mice, we gained insights into the impact of PM2.5 exposure on the health of pregnant mothers and the repercussions on the overall health of offspring when respiratory allergic inflammatory diseases are induced during pregnancy. Our study found that PM2.5 may induce inflammation in various tissues and organs of mice, extending beyond respiratory tract inflammation.

Based on the current research results, we propose that in the presence of PM2.5, the induced inflammatory response may shift from the Th2 inflammatory response to non-Th2 inflammatory mechanisms. Pathway analysis using GSEA reveals potential involvement of inflammatory responses and the TNF-α/NF-κB pathway. However, further investigation is needed to determine whether these pathways predominantly contribute to the occurrence and exacerbation of respiratory inflammatory allergic diseases or induce systemic inflammation. Additionally, exploring potential alternative mechanisms is essential.

In our study, we extensively utilized zebrafish embryo experiments. This approach allows for a more precise analysis of the impact of PM2.5 stimulation during the embryonic period on later development. It also aligns with animal welfare considerations, using species with similar but distinct responses for experimentation, thereby enhancing experimental accuracy.

GSEA results indicate that exposure to PM2.5 in mice may induce the elevation of various cytokines, which are still believed to be inflammatory responses triggered by the NF-κB pathway. Although numerous fragmentary studies continuously reveal the influence of PM2.5 on various diseases, the specific mechanisms of its effects remain unclear due to a lack of direct evidence from animal modeling experiments and even fewer embryonic experiments. Our results provide insights into the harmful effects of PM2.5 on embryos and organs. However, whether PM2.5-induced inflammatory responses in various organs and abnormal embryo development are generated through different pathways requires further study to comprehensively clarify and identify potential treatment and prevention methods.

In terms of study limitations, the composition of PM2.5 may indeed vary between batches. The current research findings demonstrate that PM2.5 obtained from the manufacturer and used in our study can indeed induce systemic inflammatory responses in experimental mice and zebrafish embryos. These findings underscore the similarity in the principal pathways activated by PM2.5 exposure, highlighting the undeniable impact of PM2.5-induced inflammation. However, in the future, we will further explore the effects of different compositions of PM2.5 on health through compositional analysis studies.

## 4. Materials and Methods

### 4.1. Mice

Wild-type female BALB/c mice aged 6 weeks and pregnant mice (National Laboratory Animal Center, Taipei, Taiwan) were used for the study. The mice were housed individually in cages with ad libitum access to food and water. All animal care and housing procedures were conducted in accordance with the guidelines of the Institutional Animal Care and Use Committee of Chung Shan Medical University (Animal Experiment Approval code: 2855).

### 4.2. Test Species and Husbandry

Wild-type (WT) AB strain zebrafish (Danio rerio) were provided by the Taiwan Zebrafish Core Facility at Academia Sinica (TZCAS) and maintained at 28 °C with a 14-h light/10-h dark cycle. Adult care and breeding were performed in compliance with relevant laws and institutional guidelines, and the Institutional Animal Care and Use Committee approved the experiments (IACUC approval no. 113021). Fish were fed three times per day with dry feed (Taikong Inc., Taipei, Taiwan). To generate embryos, adults were placed in spawning tanks in the afternoon and then spawned following the onset of light the next day. The embryos were collected and maintained in embryo media (60 μg/mL Instant Ocean^®^ (Blacksburg, VA, USA) sea salts) until the time of exposure. Embryos were staged using the criteria of Kimmel [40].

### 4.3. Protocol for PM2.5 Exposure and Challenge in Mice

PM2.5 was purchased from the Sigma-Aldrich Company, U. S. (NIST^®^ RM 8785, St. Louis, MO, USA). The PM2.5 challenge experiment lasted for a total of 28 days. Beginning with the first day of the experiment, each experimental mouse received PM2.5 stimulation through nasal inhalation every other day, resulting in a total of 14 inhalations throughout the experiment (as shown in Figure 2A). The dosage of PM2.5 administered in each inhalation was 20 μg/mL. Subsequently, on the day following the 14th PM2.5 inhalation, the mice’s airway hyperresponsiveness (AHR) was evaluated using the Buxco research system, and the animals were sacrificed after the assessment.

### 4.4. Toxin Exposure in Zebrafish

PM2.5 was purchased from the Sigma-Aldrich Company, U. S. (NIST1650B). PM2.5 was first dissolved in normal saline at a concentration of 200 mg/mL. The zebrafish embryos were exposed to vehicle (0.01 M phosphate buffered saline (PBS)) or various concentrations of PM2.5 (0, 25, 50, 100, 200, and 400 μg/mL) at 6 h post-fertilization (hpf).

### 4.5. Airway Resistance Assessment

The FinePointe RC system (Data Sciences International, DSI^TM^) was used to measure airway resistance. To establish a baseline, mice were initially challenged with aerosolized normal saline, followed by ascending doses of methacholine (METH) (0, 5, 10, and 20 mg/mL). Compliance values were recorded for a 3-min period following each challenge.

### 4.6. Preparation of Bronchoalveolar Lavage (BAL) Fluid and Analysis of Cellularity

Following the measurement of airway responsiveness, BAL was performed immediately. Mice were deeply anesthetized with 50 mg/kg of pentobarbital sodium administered intraperitoneally and euthanized by exsanguination from the abdominal aorta. The trachea was cannulated with a polyethylene tube, and the lungs were lavaged three times with 1.0 mL of physiological saline, resulting in a total fluid removal of 3.0 mL.

The BAL fluid was filtered through wet gauze (4 × 4 inches), and total cell count and viability were determined by trypan blue exclusion. The BAL fluid was then centrifuged at 150× *g* for 10 min, and the obtained pellet was suspended in 4 mL of physiological saline. Total cell counts in the BAL fluid were determined in duplicate using a hemocytometer (improved Neubauer counting chamber). Additionally, a 100 µL aliquot was centrifuged in a cytocentrifuge (model 2 Cytospin; Shandon Scientific Co., Pittsburgh, PA, USA), and differential cell counts were performed on the centrifuged preparations stained with Diff-Quick; 500 or more cells were counted for each animal at a magnification of ×1000 (oil immersion).

### 4.7. Cytokine Measurement

Cytokine and chemokine levels in serum were assessed using the Proteome Profiler Mouse XL Cytokine Array (ARY028), following the manufacturer’s protocol (R&D Systems, Inc., San Diego, CA, USA). All reagents and samples were equilibrated to room temperature prior to use, and the cytokine array kit was operated according to the manufacturer’s instructions.

### 4.8. Histological Analysis

Following the final exposure, the mice were euthanized to obtain lung tissue samples for morphological analysis. The trachea and right lung were filled with a fixative solution containing 0.8% formalin and 4% acetic acid via a ligature around the trachea. The right lung tissue was then embedded in paraffin and cut into 5 µm sections for examination. Hematoxylin and eosin (H&E) staining was performed to evaluate airway inflammation.

### 4.9. Statistical Analysis

All data were analyzed using GraphPad Prism 6 for Windows. The data are expressed as median ± IQR. For variables, the Kruskal–Wallis test (non-parametric version of ANOVA) was used for comparison. Differences with *p*-values less than 0.05 were regarded as statistically significant.

## 5. Conclusions

Our air pollution animal experimental model revealed that ambient PM2.5 can induce diverse inflammatory conditions, affirming the model’s success. Variations in the inflammatory response to PM2.5 among different populations were also evident. Additionally, our zebrafish embryo experiments provided precise insights into the embryonic impact of PM2.5, aligning with animal welfare considerations and enhancing experimental accuracy.

In pregnant mice, our studies illuminated the impact of PM2.5 exposure on maternal health and highlighted potential implications for offspring health, especially concerning respiratory allergic inflammatory diseases during pregnancy. We further confirmed PM2.5-induced inflammation across multiple tissues and organs in mice, extending beyond the respiratory tract. PM2.5 exposure may lead to elevated cytokines, indicating NF-κB pathway-triggered inflammatory responses.

While our findings contribute to understanding the harmful effects of PM2.5, elucidating the involved pathways and identifying potential treatments require further investigation, particularly in animal modeling and embryonic studies.

## Figures and Tables

**Table 1:** Illustration of the various participating genes classified through gene set enrichment analysis (GSEA) after conducting a cytokine array test on peripheral blood from experimental mice.

HALLMARK_INFLAMMATORY_RESPONSE		HALLMARK_TNFA_SIGNALING_VIA_NFKB	
PM2.5	PM2.5P	PM2.5	PM2.5P
Csf1	Cxcl5	Csf1	Cxcl5
IL12b	IL12b	IL12b	IL12b
Csf3	Csf1	Cxcl5	Csf1
Cxcl5	Ccl17	Cxcl11	Vegfa
Cxcl11	IL10	Serpine1	Cxcl2
Cxcl9	Serpine1	Ptx3	Serpine1
Serpine1	Sele	Cxcl10	Ptx3
Sele	Csf3	Vegfa	Cxcl1
Ccl17	Cxcl9	Ccl5	Il23a
Cxcl10	Ccl5	Ccl20	Ccl5
Ccl5	F3	Cxcl12	Csf2
Ccl20	Ccl22	F3	F3
Il10	Icam1	Il23a	Icam1
F3	Cxcl11	Csf2	Cxcl11
CCl22 Icam1	Cxcl10 Ccl20	Icam1 Cxcl1	Cxcl10 Ccl20

“PM2.5P” represents mice that were pregnant and exposed to PM2.5 stimulation.

## Data Availability

No new data were created or analyzed in this study. Data sharing is not applicable to this article.

## Abbreviations

AHR	airway hyperresponsiveness
BAL	bronchoalveolar lavage
BALF	bronchoalveolar lavage fluid
COPD	chronic obstructive pulmonary disease
DNA	deoxyribonucleic acid
dpf	days post-fertilization
GATA3	GATA binding protein 3
GSEA	gene set enrichment analysis
H&E	hematoxylin and eosin
hpf	hours post-fertilization
IARC	International Agency for Research on Cancer
IFN-γ	interferon gamma
IKK-α	inhibitory-κB kinase-α
IL-1β	interleukin-1 beta
IL-4	interleukin-4
IL-5	interleukin-5
IL-6	interleukin-6
IL-8	interleukin-8
IL-10	interleukin-10
IL-13	interleukin-13
TLR4	toll-like receptor 4
TLR2	toll-like receptor 2
MCP-1	monocyte chemoattractant protein-1
METH	methacholine
NC	normal control
NF-κB	nuclear factor kappa-light-chain-enhancer of activated B cells
PBS	phosphate buffered saline
PCR	polymerase chain reaction
RoRγt	RAR-related orphan receptor-γ
TNF-α	tumor necrosis factor-alpha
Th1	type 1 T helper
Th2	type 2 T helper
Th17	T helper 17 cells
Treg	regulatory T
WHO	World Health Organization
WT	wild-type
